# Supplementary material for: Multiscale Reweighted Stochastic Embedding: Deep Learning of Collective Variables for Enhanced Sampling
Source: J Phys Chem A. 2021 Jul 2;125(28):6286–302. doi: 10.1021/acs.jpca.1c02869 (PMC8389995; doi:10.1021/acs.jpca.1c02869)
Supplement: Supplementary file 1 — jp1c02869_si_001.pdf [file jp1c02869_si_001.pdf]

Supporting Information: Multiscale Reweighted Stochastic Embedding (MRSE): Deep Learning of Collective Variables for Enhanced Sampling

Jakub Rydzewski*,† and Omar Valsson*,‡

†*Institute of Physics, Faculty of Physics, Astronomy and Informatics, Nicolaus Copernicus University, Grudziadzka 5, 87-100 Torun, Poland*

‡*Max Planck Institute for Polymer Research, Ackermannweg 10, D-55128 Mainz, Germany*

E-mail: jr@fizyka.umk.pl; valsson@mpip-mainz.mpg.de

Contents

S1 Entropy of the Reweighted Feature Pairwise Probability Distribution S-3

S2 Kullback-Leibler Divergence Loss for a Full Set of Training Data S-5

S3 Description of Well-Tempered Farthest-Point Sampling (WT-FPS) S-7

S4 Effective Landmark CV Distribution for Weight-Tempered Random Sampling S-8

S5 Details About Clustering Used in Figure 7 . . . . S-10

S6 Bandwidth Values for Kernel Density Estimation S-11

S7 Loss Function Learning Curves S-12

S8 Additional Embeddings for the Müller-Brown Potential S-13

S9 Feature Preprocessing in the Alanine Dipeptide System S-14

S10 Alanine Dipeptide Embeddings for Different Values of α in Weight-Tempered Random Sampling S-15

S11 Alanine Dipeptide Embeddings for $\alpha = 2$ in WT-FPS S-16

S12 Alanine Dipeptide Embeddings for Different Random Seed Values S-17

S13 Convergence of Alanine Tetrapeptide Simulations S-18

References **S-21**

S1 Entropy of the Reweighted Feature Pairwise Probability Distribution

Here, we derive eq 11 and eq 12 from Section 2.2.2. The Shannon entropy of a discrete Gaussian kernel is:

$$H(\mathbf{x}_i) = -\sum_j p_{ij}^{\epsilon_i} \log p_{ij}^{\epsilon_i}, \tag{S1}$$

where $p_{ij}^{\epsilon_i}$ are matrix elements of the ith row of the reweighted feature probability distribution:

$$p_{ij}^{\boldsymbol{\epsilon}} = \frac{\tilde{K}_{\epsilon_i}(\mathbf{x}_i, \mathbf{x}_j)}{\sum_k \tilde{K}_{\epsilon_k}(\mathbf{x}_i, \mathbf{x}_k)}, \tag{S2}$$

for the reweighted Gaussian kernel $\tilde{K}_{\epsilon_i}(\mathbf{x}_i, \mathbf{x}_j) = r(\mathbf{x}_i, \mathbf{x}_j) K_{\epsilon_i}(\mathbf{x}_i, \mathbf{x}_j)$, where $r(\mathbf{x}_i, \mathbf{x}_j)$ is a reweighting factor. We define a row-wise normalization constant of eq S2 as $\bar{p}_i = \sum_k \tilde{K}_{\epsilon_i}(\mathbf{x}_i, \mathbf{x}_k)$. By plugging eq S2 to eq S1, we arrive at:

$$\begin{aligned}
H(\mathbf{x}_i) &= -\frac{1}{\bar{p}_i} \sum_j \tilde{K}_{\epsilon_i}(\mathbf{x}_i, \mathbf{x}_j) \log \left(\frac{1}{\bar{p}_i} \tilde{K}_{\epsilon_i}(\mathbf{x}_i, \mathbf{x}_j) \right) \\
&= -\frac{1}{\bar{p}_i} \sum_j \tilde{K}_{\epsilon_i}(\mathbf{x}_i, \mathbf{x}_j) \log \left(\frac{1}{\bar{p}_i} r(\mathbf{x}_i, \mathbf{x}_j) K_{\epsilon_i}(\mathbf{x}_i, \mathbf{x}_j) \right) \\
&= -\frac{1}{\bar{p}_i} \sum_j \tilde{K}_{\epsilon_i}(\mathbf{x}_i, \mathbf{x}_j) \Big(\log r(\mathbf{x}_i, \mathbf{x}_j) + \log K_{\epsilon_i}(\mathbf{x}_i, \mathbf{x}_j) - \log \bar{p}_i \Big) \\
&= -\frac{1}{\bar{p}_i} \sum_j \tilde{K}_{\epsilon_i}(\mathbf{x}_i, \mathbf{x}_j) \Big(\log r(\mathbf{x}_i, \mathbf{x}_j) - \epsilon_i \|\mathbf{x}_i - \mathbf{x}_j\|_2^2 - \log \bar{p}_i \Big),
\end{aligned} \tag{S3}$$

which further translates to:

$$H(\mathbf{x}_i) = \frac{1}{\bar{p}_i}\Big(\epsilon_i \sum_j \tilde{K}_{\epsilon_i}(\mathbf{x}_i, \mathbf{x}_j)\|\mathbf{x}_i - \mathbf{x}_j\|_2^2$$
$$- \sum_j \tilde{K}_{\epsilon_i}(\mathbf{x}_i, \mathbf{x}_j) \log r(\mathbf{x}_i, \mathbf{x}_j)$$
$$+ \log \bar{p}_i \underbrace{\sum_j \tilde{K}_{\epsilon_i}(\mathbf{x}_i, \mathbf{x}_j)}_{=\bar{p}_i}\Big), \tag{S4}$$

and finally, the entropy of the reweighted feature probability distribution is given by:

$$\begin{aligned} H(\mathbf{x}_i) &= \frac{1}{\bar{p}_i}\Big(\epsilon_i \sum_j \tilde{K}_{\epsilon_i}(\mathbf{x}_i, \mathbf{x}_j)\|\mathbf{x}_i - \mathbf{x}_j\|_2^2 - \sum_j \tilde{K}_{\epsilon_i}(\mathbf{x}_i, \mathbf{x}_j) \log r(\mathbf{x}_i, \mathbf{x}_j)\Big) + \log \bar{p}_i \\ &= \frac{1}{\bar{p}_i}\epsilon_i \sum_j \tilde{K}_{\epsilon_i}(\mathbf{x}_i, \mathbf{x}_j)\|\mathbf{x}_i - \mathbf{x}_j\|_2^2 \underbrace{- \frac{1}{\bar{p}_i} \sum_j \tilde{K}_{\epsilon_i}(\mathbf{x}_i, \mathbf{x}_j) \log r(\mathbf{x}_i, \mathbf{x}_j)}_{H_V(\mathbf{x}_i)} + \log \bar{p}_i. \end{aligned} \tag{S5}$$

The full Shannon entropy vector is $H = [H(\mathbf{x}_1), H(\mathbf{x}_2), \ldots, H(\mathbf{x}_N)]^T$.

If weights are of exponential form (like in WT-MetaD), $w(\mathbf{x}_i) = \mathrm{e}^{\beta V(\mathbf{x}_i)}$, then $H_V(\mathbf{x}_i)$ may be further reduced:

$$\begin{aligned} H_V(\mathbf{x}_i) &= -\frac{1}{\bar{p}_i} \sum_j \tilde{K}_{\epsilon_i}(\mathbf{x}_i, \mathbf{x}_j) \log r(\mathbf{x}_i, \mathbf{x}_j) \\ &= -\frac{1}{\bar{p}_i} \sum_j \tilde{K}_{\epsilon_i}(\mathbf{x}_i, \mathbf{x}_j) \log \sqrt{\mathrm{e}^{\beta V(\mathbf{x}_i)} \mathrm{e}^{\beta V(\mathbf{x}_j)}} \\ &= -\frac{\beta}{2\bar{p}_i} \sum_j \tilde{K}_{\epsilon_i}(\mathbf{x}_i, \mathbf{x}_j) \left(V(\mathbf{x}_i) + V(\mathbf{x}_j)\right) \\ &= -\frac{\beta}{2} \left(\frac{1}{\bar{p}_i} \sum_j \tilde{K}_{\epsilon_i}(\mathbf{x}_i, \mathbf{x}_j) V(\mathbf{x}_j) + V(\mathbf{x}_i) \right). \end{aligned} \tag{S6}$$

S2 Kullback-Leibler Divergence Loss for a Full Set of Training Data

Here, we present a derivation of the Kullback-Leibler (KL) divergence between two pairwise probability distributions:

$$\mathbf{M} = \Big(p_{ij}\Big)_{1\leq i,j\leq N} \quad \text{and} \quad p_{ij} = \frac{1}{N_P}\sum_{P}^{N_P} p_{ij}^{\boldsymbol{\epsilon}^P} \tag{S7}$$

and

$$\mathbf{Q} = \Big(q_{ij}\Big)_{1\leq i,j\leq N} \quad \text{and} \quad q_{ij} = \frac{\left(1+\|\mathbf{s}_i - \mathbf{s}_j\|_2^2\right)^{-1}}{\sum_k \left(1+\|\mathbf{s}_i - \mathbf{s}_k\|_2^2\right)^{-1}}, \tag{S8}$$

as is described in Sections 2.2.3 and 2.2.4 in the main text.

For discrete probability distributions p and q defined on the same probability space χ, the KL divergence of p and q is defined as:

$$D_{\mathrm{KL}}(p\,||\,q) = \sum_{x\in\chi} p(x)\log\left(\frac{p(x)}{q(x)}\right), \tag{S9}$$

where $q(x) = 0$ implies $p(x) = 0$, and the base of the logarithm is arbitrary. For pairwise probability matrices, eq S9 needs to be rewritten as the row-wise average of the KL divergences:

$$D_{\mathrm{KL}}\big(\mathbf{M}\|\mathbf{Q}\big) = \frac{1}{N_b}\sum_{i=1}^{N_b} D_{\mathrm{KL}}(\mathbf{M}_i\,||\,\mathbf{Q}_i), \tag{S10}$$

where $\mathbf{M}_i$ and $\mathbf{Q}_i$ are the ith rows of $\mathbf{M}$ and $\mathbf{Q}$, respectively, and N_b is the number of rows in the matrices or the batch size. Then, if we take a sum of the partial KL divergence losses for all B batches of the data using eq S10, we arrive at the KL divergence for a full set of the

training data:

$$\begin{aligned} D_{\mathrm{KL}}\big(\mathbf{M}\|\mathbf{Q}\big) &= \sum_{b=1}^{B} \frac{1}{N_b} \sum_{i=1}^{N_b} \sum_{\substack{j=1 \\ i\neq j}}^{N_b} p_{ij} \log\left(\frac{p_{ij}}{q_{ij}}\right) \\ &= \frac{1}{N_b} \sum_{b=1}^{B} \sum_{i=1}^{N_b} \sum_{\substack{j=1 \\ i\neq j}}^{N_b} p_{ij} \log\left(\frac{p_{ij}}{q_{ij}}\right). \end{aligned} \tag{S11}$$

S3 Description of Well-Tempered Farthest-Point Sampling (WT-FPS)

Well-tempered farthest-point sampling[S1] (WT-FPS) bridges two selection landmark selection schemes, weighted random sampling[S2] and farthest point sampling (FPS).[S3] Random sampling with weights should, in principle, give an equilibrium representation.[S1] However, there might be issues with capturing metastable states lying higher in free energy. FPS ignores the underlying data density and results in a subset that is most broadly spread out in the feature space.

In WT-FPS, we select N landmarks from a dataset that consists of M feature samples. We start by selecting $K = \sqrt{NM}$ samples using FPS, and treat them as the centers of a Voronoi diagram. The remaining samples are assigned to their nearest center. Next, we assign a weight to each center, which is obtained by summing over the weights of all the samples that belong to a center, $\omega_i = \sum_k w(\mathbf{x}_k)$, where i is the label of a center. We define a weight of the ith center as:

$$d_i = \left(\frac{\omega_i}{\sum_{j=1}^{K} \omega_j} \right)^{1/\alpha}, \tag{S12}$$

where we introduce a broadening parameter α as in the well-tempered distribution. To select the landmarks, we proceed by randomly selecting a center according to its weight d_i. We then choose at random a feature sample from this center (taking in account the weights of feature samples $w(\mathbf{x}_k)$). We repeat this procedure until we have N landmarks.

If $\alpha = 1$, we recover weighted random sampling. However, by choosing $\alpha > 1$, we increasingly favor the algorithm to ignore the underlying probabilities and pick a more uniformly distributed set of landmarks. In the limit of $\alpha \to \infty$, we obtain FPS. Thus, by using $\alpha > 1$ that is not too large, we can obtain a landmark set that also represents higher-lying metastable states while not being far from the equilibrium distribution.

S4 Effective Landmark CV Distribution for Weight-Tempered Random Sampling

Starting from eq 16 in the main manuscript:

$$P_\alpha(\mathbf{x}) = \frac{\int \mathrm{d}\mathbf{R}\, [w(\mathbf{R})]^{1/\alpha}\, \delta\left[\mathbf{x} - \mathbf{x}(\mathbf{R})\right] P_V(\mathbf{R})}{\int \mathrm{d}\mathbf{R}\, [w(\mathbf{R})]^{1/\alpha}\, P_V(\mathbf{R})} \tag{S13}$$

for the landmark feature distribution in weight-tempered random sampling, we want to derive the distribution in CV space, $P_\alpha(\mathbf{s})$.

Setting $\mathbf{x} = \mathbf{s}$, and assuming that the weights are given by $w(\mathbf{R}) = \mathrm{e}^{\beta V(\mathbf{s}(\mathbf{R}))}$ (i.e., come from a CV-based enhanced sampling method), we obtain:

$$\begin{aligned} P_\alpha(\mathbf{s}) &\propto \int \mathrm{d}\mathbf{R}\, \left[\mathrm{e}^{\beta V(\mathbf{s}(\mathbf{R}))}\right]^{1/\alpha} \delta\left[\mathbf{s} - \mathbf{s}(\mathbf{R})\right] P_V(\mathbf{R}) \\ &= \int \mathrm{d}\mathbf{R}\, \mathrm{e}^{\frac{1}{\alpha}\beta V(\mathbf{s})} \delta\left[\mathbf{s} - \mathbf{s}(\mathbf{R})\right] P_V(\mathbf{R}) \\ &= \mathrm{e}^{\frac{1}{\alpha}\beta V(\mathbf{s})} \int \mathrm{d}\mathbf{R}\, \delta\left[\mathbf{s} - \mathbf{s}(\mathbf{R})\right] P_V(\mathbf{R}) \\ &= \mathrm{e}^{\frac{1}{\alpha}\beta V(\mathbf{s})} P_V(\mathbf{s}), \end{aligned} \tag{S14}$$

where in the first step we use the property of the delta function to sift out the dependence of $\mathbf{R}$ in $\mathrm{e}^{\frac{1}{\alpha}\beta V(\mathbf{s})}$, and in the last step the definition of $P_V(\mathbf{s})$ from eq 2 in the main manuscript. As $P_V(\mathbf{s}) \propto P(\mathbf{s})\mathrm{e}^{-\beta V(\mathbf{s})}$, we obtain two equivalent ways of writing $P_\alpha(\mathbf{s})$, using either the equilibrium distribution $P(\mathbf{s})$ or the biased distribution $P_V(\mathbf{s})$:

$$\begin{aligned} P_\alpha(\mathbf{s}) &= \frac{\mathrm{e}^{\frac{1}{\alpha}\beta V(\mathbf{s})} P_V(\mathbf{s})}{\int \mathrm{d}\mathbf{s}\, \mathrm{e}^{\frac{1}{\alpha}\beta V(\mathbf{s})} P_V(\mathbf{s})} \\ &= \frac{\mathrm{e}^{\left(\frac{1}{\alpha}-1\right)\beta V(\mathbf{s})} P(\mathbf{s})}{\int \mathrm{d}\mathbf{s}\, \mathrm{e}^{\left(\frac{1}{\alpha}-1\right)\beta V(\mathbf{s})} P(\mathbf{s})}. \end{aligned} \tag{S15}$$

These are the equations for a general CV-based bias potential.

In the case of WT-MetaD, the bias potential at convergence is given by $V(\mathbf{s}) = -\left(1 - \frac{1}{\gamma}\right) F(\mathbf{s})$

(eq 3 in the main manuscript). Inserting this for $V(\mathbf{s})$ and using $P(\mathbf{s}) \propto \mathrm{e}^{-\beta F(\mathbf{s})}$ leads to:

$$\begin{aligned} P_\alpha(\mathbf{s}) &\propto \mathrm{e}^{-\left(\frac{1}{\alpha}-1\right)\left(1-\frac{1}{\gamma}\right)F(\mathbf{s})}\mathrm{e}^{-\beta F(\mathbf{s})} \\ &= \mathrm{e}^{-\left(\frac{1}{\alpha}-\frac{1}{\alpha}\frac{1}{\gamma}+\frac{1}{\gamma}\right)F(\mathbf{s})} \\ &= \left[\mathrm{e}^{-\beta F(\mathbf{s})}\right]^{\left(\frac{1}{\alpha}-\frac{1}{\alpha}\frac{1}{\gamma}+\frac{1}{\gamma}\right)} \end{aligned} \tag{S16}$$

or

$$\begin{aligned} P_\alpha(\mathbf{s}) &= \frac{[P(\mathbf{s})]^{\left(\frac{1}{\alpha}-\frac{1}{\alpha}\frac{1}{\gamma}+\frac{1}{\gamma}\right)}}{\int \mathrm{d}\mathbf{s}\ [P(\mathbf{s})]^{\left(\frac{1}{\alpha}-\frac{1}{\alpha}\frac{1}{\gamma}+\frac{1}{\gamma}\right)}} \\ &= \frac{[P(\mathbf{s})]^{1/\tilde{\alpha}}}{\int \mathrm{d}\mathbf{s}\ [P(\mathbf{s})]^{1/\tilde{\alpha}}}, \end{aligned} \tag{S17}$$

where we introduce an effective tempering parameter $\tilde{\alpha}$ as:

$$\tilde{\alpha} = \left(\frac{1}{\alpha} - \frac{1}{\alpha\gamma} + \frac{1}{\gamma}\right)^{-1} = \frac{\gamma\alpha}{\gamma+\alpha-1}. \tag{S18}$$

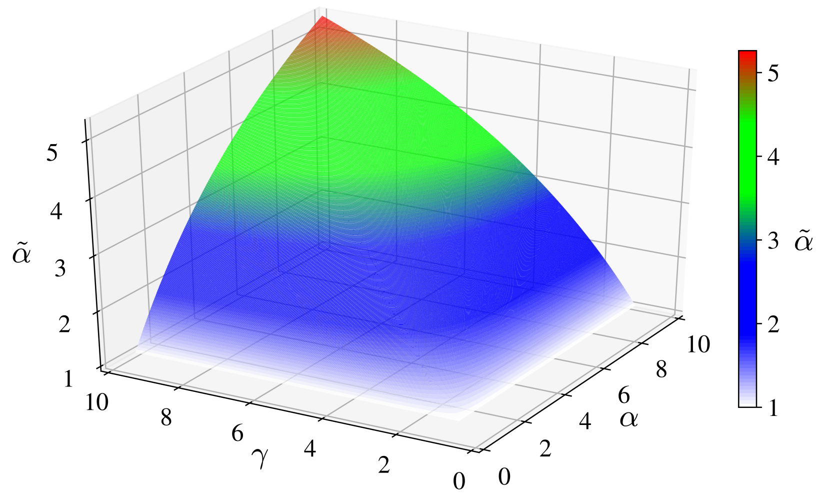

Figure S1: Effective alpha $\tilde{\alpha}$ as a function of α and γ.

S5 Details About Clustering Used in Figure 7

We perform the cluster analysis on the MRSE embedding shown in Figure 7 by fitting a Bayesian Gaussian mixture to the data. We run this procedure with the number of components (clusters) equal to 3, where each component has its own general covariance matrix. The clustering takes the periodicity of (Φ, Ψ) into account. We use the `scikit-learn` machine-learning library[S4] to perform this analysis.

S6 Bandwidth Values for Kernel Density Estimation

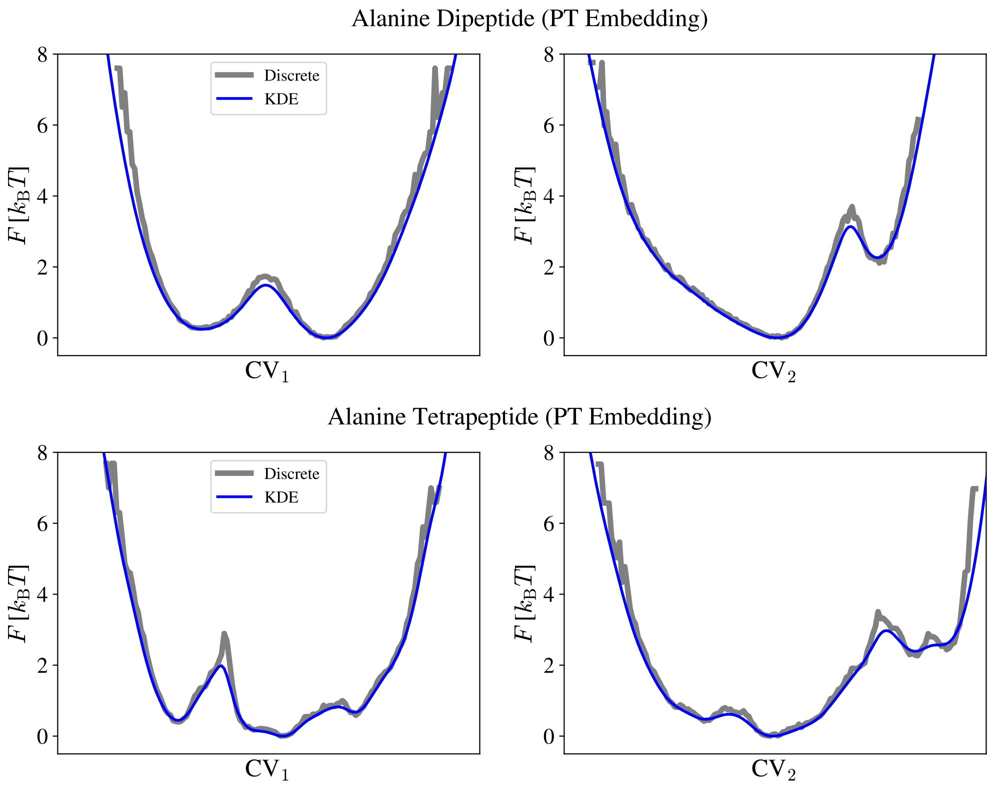

Figure S2: Free energy profiles for the PT embeddings of alanine dipeptide (top row) and alanine tetrapeptide (bottom row). The profiles are obtained using using histogramming ("Discrete", black lines) using 200 grid points and kernel density estimation ("KDE", blue lines) using bandwidth values estimated based on Silverman's rule (see Section 3.4).

S7 Loss Function Learning Curves

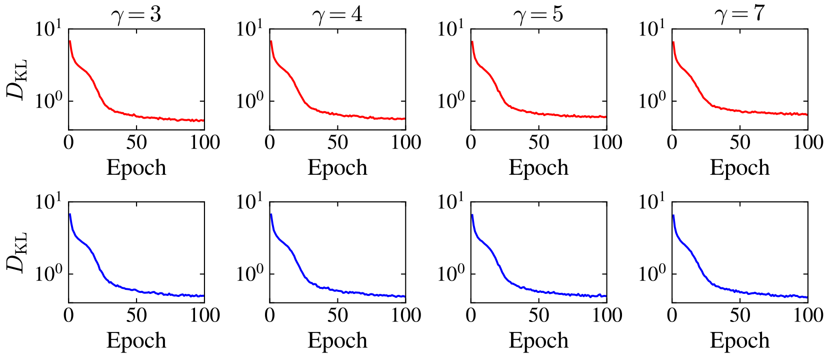

Figure S3: Results for the Müller-Brown potential (Section 4.1). Learning curves from the minimization of the Kullback-Leibler loss function for the MRSE embeddings shown in Figure S1. The upper row shows results obtained by incorporating weights into the training (red lines, labeled reweighted in Figure S1), while the lower row shows results obtained by not incorporating weights into the training (blue lines, labeled not reweighted in Figure S1). The y-axis is shown as a logarithmic (base 10) scale.

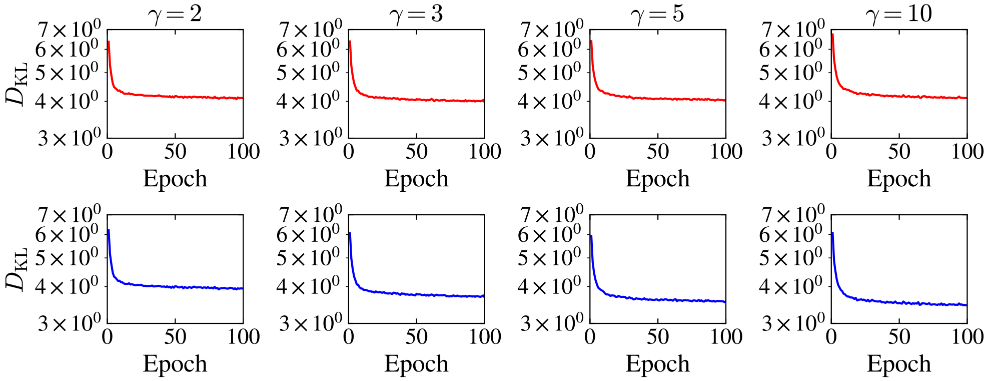

Figure S4: Results for alanine dipeptide in vacuum at 300 K (Section 4.2). Learning curves from the minimization of the Kullback-Leibler loss function for the MRSE embeddings shown in Figure 7(c) in the main paper. The upper row shows results obtained by incorporating weights into the training (red lines, labeled reweighted in Figure 7(c)), while the lower row shows results obtained by not incorporating weights into the training (blue lines, labeled not reweighted in Figure 7(c)). The y-axis is shown as a logarithmic (base 10) scale.

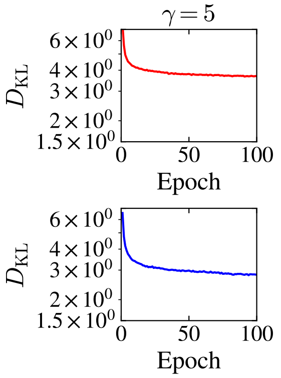

Figure S5: Results for alanine tetrapeptide in vacuum at 300 K (Section 4.3). Learning curves from the minimization of the Kullback-Leibler loss function for the MRSE embeddings shown in Figure 11(b-c) in the main paper. The upper row shows results obtained by incorporating weights into the training (red lines, labeled reweighted in Figure 11(c)), while the lower row shows results obtained by not incorporating weights into the training (blue lines, labeled not reweighted in Figure 11(b)). The y-axis is shown as a logarithmic (base 10) scale.

S8 Additional Embeddings for the Müller-Brown Potential

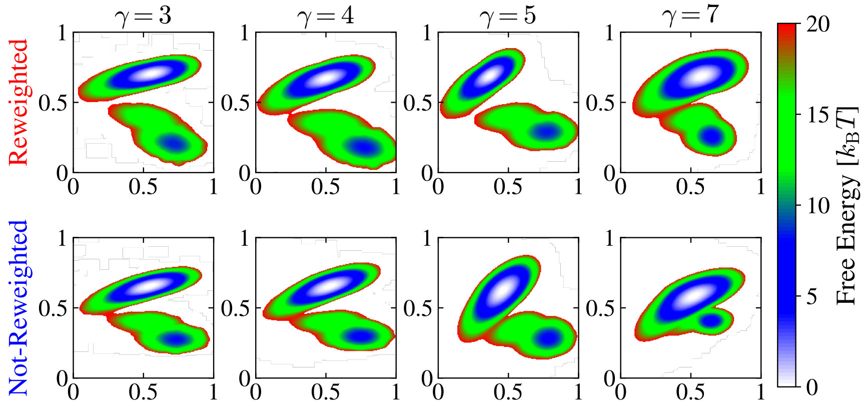

Figure S6: Results for the Müller-Brown potential (Section 4.1). FESs for the MRSE embeddings obtained from biased simulation data. We show results obtained from WT-MetaD simulations using different bias factors γ (3, 4, 5, 7). The upper row (reweighted) shows results obtained by incorporating weights into the training via a reweighted feature pairwise probability distribution, while the lower row shows results obtained by not incorporating weights (not reweighted). The embeddings are not rotated using the Procrustes algorithm to fit to the MB potential, unlike those from Figure 4 in the main text. The units for the MRSE embeddings are arbitrary and thus not shown.

S9 Feature Preprocessing in the Alanine Dipeptide System

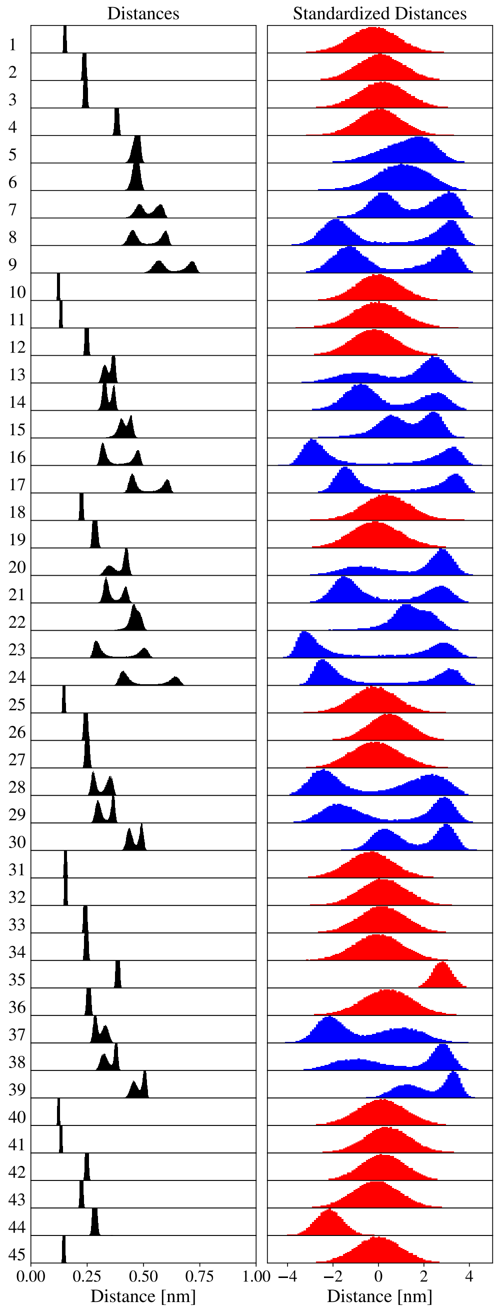

Figure S7: Results for alanine dipeptide in vacuum at 300 K (Section 4.2). Heavy-atom distances used as the input features for the MRSE embeddings. The first column shows the histograms of the unprocessed distances (black), while the second column depicts standardized distances. We discarded all distances with a variance below 2×10^{-4} nm^2 from the training set (shown in red). We can see that the remaining 21 distances (shown in blue) used for the training are mostly binomial.

S10 Alanine Dipeptide Embeddings for Different Values of α in Weight-Tempered Random Sampling

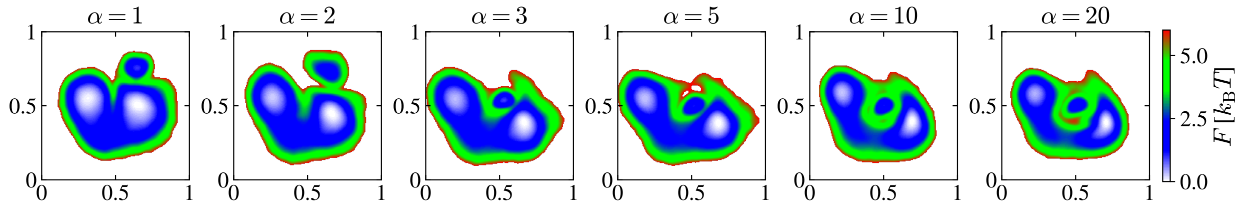

Figure S8: Results for alanine dipeptide in vacuum at 300 K (Section 4.2). FESs for the MRSE embedding obtained from a WT-MetaD simulation data ($\gamma = 5$), where we use the weight-tempered random sampling for landmark selection with $\alpha = \{1, 2, 3, 5, 10, 20\}$ (see Figure 5). In the cases for $\alpha > 2$, we obtain inadequate mappings of the metastable states. The units for the MRSE embeddings are arbitrary and thus not shown.

S11 Alanine Dipeptide Embeddings for $\alpha = 2$ in WT-FPS

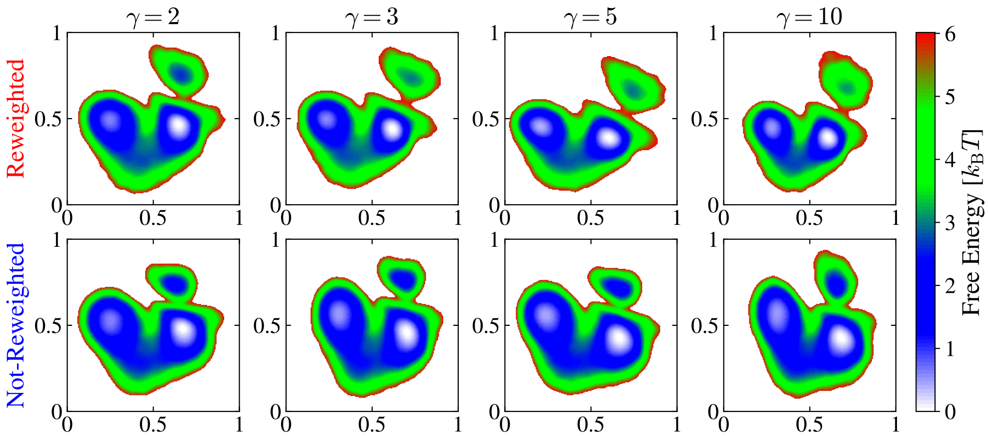

Figure S9: Results for alanine dipeptide in vacuum at 300 K (Section 4.2). FESs for the MRSE embedding obtained from a WT-MetaD simulation data, where we use the WT-FPS landmark selection with $\alpha = 2$. The units for the MRSE embeddings are arbitrary and thus not shown.

S12 Alanine Dipeptide Embeddings for Different Random Seed Values

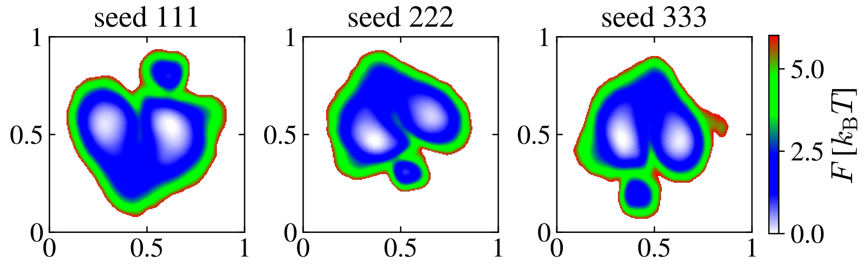

Figure S10: Results for alanine dipeptide in vacuum at 300 K (Section 4.2). FESs for MRSE embeddings trained using the PT simulation data obtained using different random seed values. The the seed affects both the landmark selection and the shuffling of the landmarks during the training. The embeddings differ mostly in how they are rotated. The units for the MRSE embeddings are arbitrary and thus not shown.

S13 Convergence of Alanine Tetrapeptide Simulations

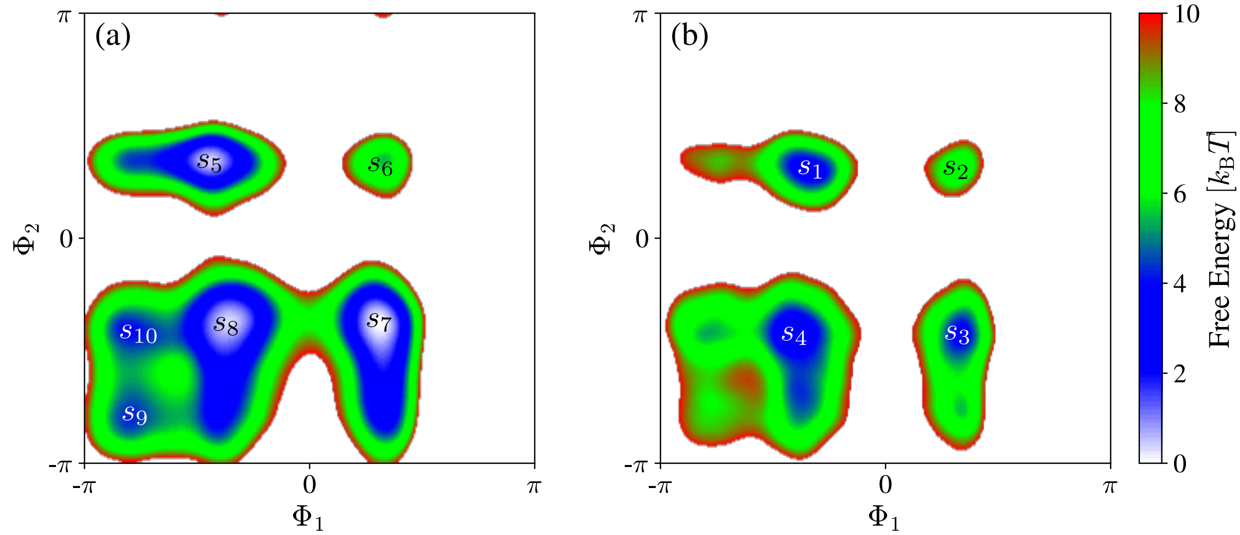

Figure S11: Results for alanine tetrapeptide in vacuum at 300 K (Section 4.3). The conditional FESs (eq 17) as a function of Φ_1 and Φ_2 for the (a) A and (b) B minima of Φ_3 shown in Figure 10(c) in the main paper. These are the same conditional FESs as shown in Figure 10(a).

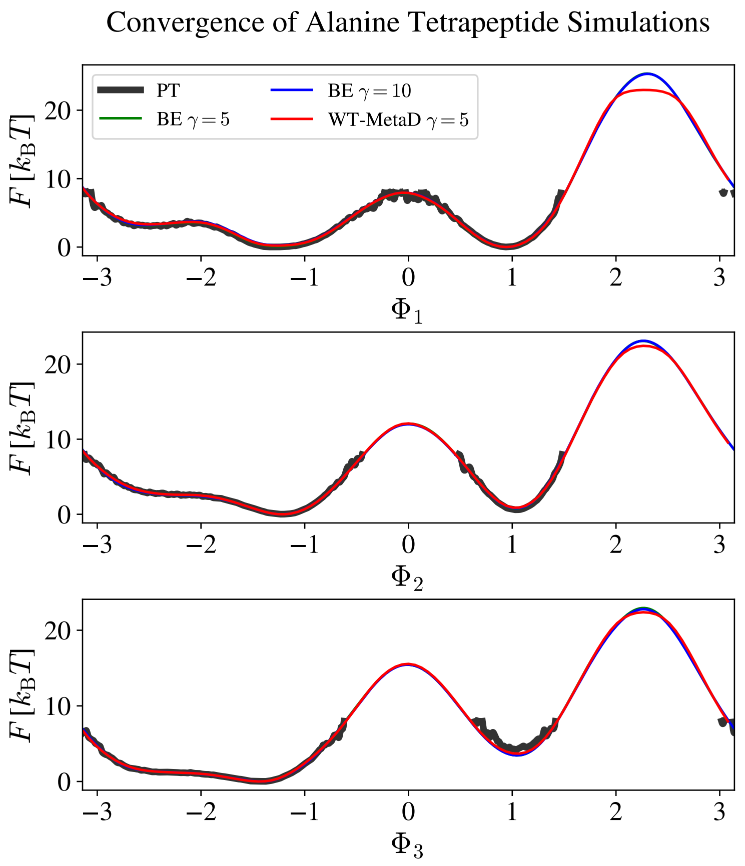

Figure S12: Results for alanine tetrapeptide in vacuum at 300 K (Section 4.3). Free energy profiles for the $\mathbf{\Phi}$ dihedral angles. We show free energy profiles for the PT simulation (black lines), the WT-MetaD simulation ($\gamma = 5$, red line) and two bias-exchange metadynamics simulations ($\gamma = 5$ and $\gamma = 10$, green and blue, respectively). The free energy profiles for the WT-MetaD and the bias-exchange simulations are obtained by summing up the Gaussians using `sum_hills` command line tool in PLUMED. The free energy profiles for the PT simulation are obtained using histogramming with 200 grid points. In the bias-exchange simulations, we employ 6 replicas each one biasing one of the dihedral angles $(\mathbf{\Phi}, \mathbf{\Psi})$ using WT-MetaD with bias factors 5 or 10. We attempt exchanges between neighboring replicas every 10 ps.

As expected, the PT simulation does not capture the higher-lying regions. We can see that all the simulations agree well for the minima. Furthermore, the WT-MetaD and bias-exchange simulations are in good agreement for the whole curve apart from the highest barrier regions. Therefore, we conclude that the WT-MetaD and PT simulations are converged.

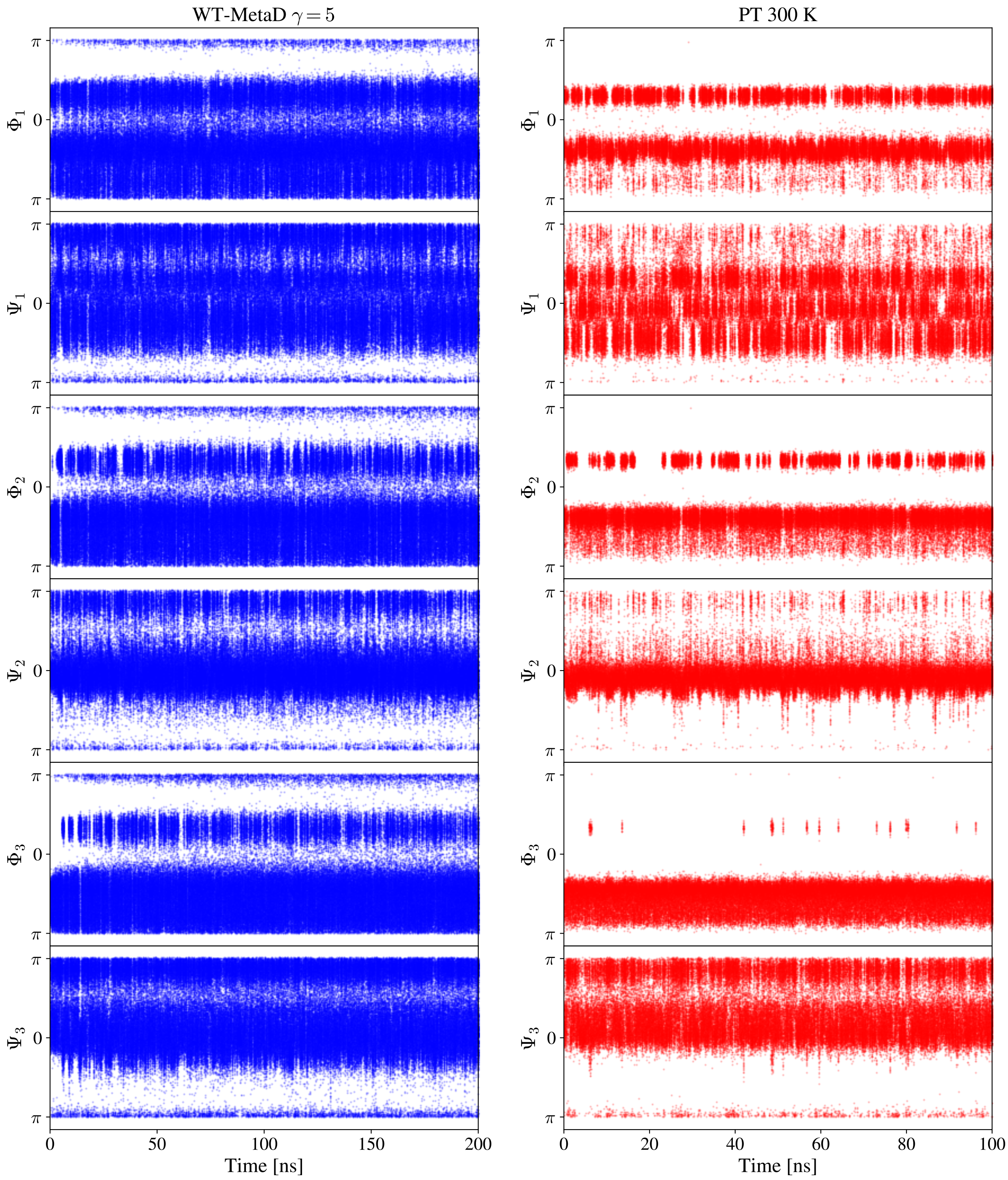

Figure S13: Results for alanine tetrapeptide in vacuum at 300 K (Section 4.3). Time series of the dihedral angles for the WT-MetaD simulations ($\gamma = 5$) biasing $\mathbf{\Phi}$ and for the 300 K replica in the PT simulation.

References

(S1) Ceriotti, M.; Tribello, G. A.; Parrinello, M. Demonstrating the transferability and the descriptive power of sketch-map. *J. Chem. Theory Comput.* **2013**, *9*, 1521–1532.

(S2) Tribello, G. A.; Gasparotto, P. *Biomolecular Simulations: Methods and protocols*; Springer, 2019; p 453.

(S3) Hochbaum, D. S.; Shmoys, D. B. A best possible heuristic for the *k*-center problem. *Math. Oper. Res.* **1985**, *10*, 180–184.

(S4) Pedregosa, F.; Varoquaux, G.; Gramfort, A.; Michel, V.; Thirion, B.; Grisel, O.; Blondel, M.; Prettenhofer, P.; Weiss, R.; Dubourg, V.; Vanderplas, J.; Passos, A.; Cournapeau, D.; Brucher, M.; Perrot, M.; Duchesnay, E. scikit-learn: Machine learning in Python. *J. Mach. Lear. Res.* **2011**, *12*, 2825–2830.
